# An Evaluation of Policies Promoting Physical Activity among Filipino Youth

**DOI:** 10.3390/ijerph20042865

**Published:** 2023-02-06

**Authors:** Yves Y. Palad, Roselle M. Guisihan, Maria Eliza R. Aguila, Ralph Andrew A. Ramos, Jonathan Y. Cagas

**Affiliations:** 1 Department of Physical Therapy, College of Allied Medical Professions, University of the Philippines Manila, Manila 1000, Philippines; 2 Department of Sports Science, College of Human Kinetics, University of the Philippines Diliman, Quezon 1101, Philippines

**Keywords:** active lifestyle, disease prevention, government indicator, health promotion, lower middle-income country, sedentary behavior

## Abstract

Government is key to promoting physical activity at the population level through policy. The government was one of the physical activity indicators graded for the 2022 Philippine Physical Activity Report Card based on ten physical activity-related policies. This study aimed to evaluate the scope of the policies and to update these policies. Philippine government databases were searched for policies using physical activity-related key terms. Policies found were evaluated using the Wales Active Healthy Kids scoring rubric. The overall grade was converted to a letter grade based on the Global Matrix 4.0 grading system. The authors analyzed the policies’ scope and implications to practice and policy. Seven additional policies were found. Considering all 17 policies, the government indicator grade is now A- from the preliminary grade of B. The scope covers promoting physical activity mainly through sports participation and active transport among students, student-athletes, persons with disabilities, and the general population in school and community settings. The gap between government and overall physical activity (F) scores suggests the need for a comprehensive physical activity plan promoting various forms of physical activity and reducing sedentary behavior among all Filipino youth and across various settings. Crucial to achieving change is a well-coordinated, whole-of-systems approach to promoting active healthy lifestyles.

## 1. Introduction

An active healthy lifestyle among children and adolescents is vital to their overall development as it improves their physical functioning, mental and physical health, and mitigates health risks [1]. Having an active healthy lifestyle includes engaging in recommended levels of physical activity and reducing sedentary times [1]. For children and adolescents, the World Health Organization (WHO) recommends at least an average of 60 min of moderate-to-vigorous physical activity per day and reduced sedentary behaviors, particularly recreational screen time use [1]. Early engagement in appropriate physical activity levels is important as it is directly associated with physical activity [2] and better perceptions of health in adulthood [3].

Such an active healthy lifestyle is essential in preventing non-communicable diseases (NCDs) [4]. They are chronic diseases resulting from a combination of genetic, physiological, environmental, and behavioral factors including physical inactivity, and are the leading causes of death globally [5]. A recent report from the WHO revealed that globally, there will be about 500 million new NCD cases in the next ten years, costing the world around USD 27 billion if not managed [4]. Almost three-quarters of the new NCD cases will be in lower and upper middle-income countries [4]. Having a physically active populace is, therefore, beneficial in curbing the economic burden of NCDs [4].

The Philippines, being a lower middle-income country, is one of these countries that will bear much of the economic burden from rising NCD cases. The country, therefore, needs to scale up its efforts to promote an active healthy lifestyle, especially since its youth was found to be insufficiently physically active [6]. As of 2020, 10% of the Philippine population, or 11 million, are children below 5 years, and 39%, or 42 million, are 5 to 24 years old [7]. Despite the importance of adopting an active healthy lifestyle, children and adolescents were found to be less active than might be expected [4]. According to a national survey conducted from January 2018 to February 2019, 82.7% of 10- to 17-year-old Filipino adolescents were not meeting physical activity recommendations [6,8]. These data were gathered from approximately 46,000 households across the country [8].

The national government is a key element in a coordinated, whole-of-systems approach to scaling up physical activity promotion efforts at the population level [4,9,10]. The WHO Global Action Plan on Physical Activity 2018–2030 (GAPPA) suggests that strong commitment and leadership are needed to establish a national vision and direction that prioritize the promotion of physical activity and reducing sedentary behaviors [10]. The government is a key source of such leadership and commitment through policymaking that promotes actions toward a more active and healthy population, and through resource mobilization to support a coordinated, multi-sectoral policy implementation, monitoring, evaluation, and reporting [10].

Government is also one of the ten indicators of success in the Global Matrix 4.0 (see Table 1 for these ten indicators and their definitions). The Global Matrix 4.0 is an initiative that aims to evaluate the status of countries in promoting physical activity participation among the youth based on synthesized available data on each of the ten indicators. Each indicator is assigned a letter grade (i.e., A to F, INC) based on how well a country performed against the indicator’s benchmarks [11] Table 2 shows the Global Matrix 4.0 grading rubric. Benchmarks for evaluating the government indicator include “evidence of leadership and commitment in providing physical activity opportunities for all children and youth”, “allocated funds and resources for the implementation of physical activity promotion strategies and initiatives for all children and youth”, and “demonstrated progress through the key stages of public policy making” [11]. Participating countries in the Global Matrix 4.0 were graded a C on average for their government efforts in influencing the physical activity of children and adolescents [11].

The Philippines was one of the countries that participated in the Global Matrix 4.0 and produced its own physical activity country report card following the Global Matrix 4.0 recommended methodology [12]. A working research group was formed to work on the Report Card. The group was composed of 25 academics and practitioners in the fields of physical activity, sports science, physical therapy, physical education, and psychology [12]. Each member was assigned to an indicator and was responsible for searching databases for documents on the physical activity of Filipino youth, extracting information from obtained documents, evaluating the assigned indicator against its benchmarks, and drafting the Report Card.

Two members of the research working group were assigned to lead the evaluation of the government indicator. It was graded a B based on the assessment of ten (10) national policies that promote any form of physical activity (e.g., organized sports, active transport, recreational play, etc.). These were obtained from searching government websites including those of the Congress and Senate of the Philippines, the Department of Health, the Department of Education, the Philippine Sports Commission, the National Council for Disability Affairs, the Department of Budget and Management, the Housing and Land Use Regulatory Board, and the Official Gazette of the Republic of the Philippines.

The policy scoring rubric developed by the Wales Active Healthy Kids (AHK-Wales) group [13] was used to quantitatively evaluate the policies. The rubric consists of six criteria based on the second version of the Health-Enhancing Physical Activity Policy Audit Tool developed by the WHO Regional Office for Europe [13]. Table 3 outlines the general description of each criterion. A full description of the AHK-Wales scoring system is available in the article by Ward et al. [13]. The overall score was then converted to a letter grade based on the Global Matrix 4.0 grading rubric [11]. Stakeholders from government agencies, education institutions, professional organizations, and non-government organizations relevant to the physical activity of youth were consulted about preliminary results. Government indicator scores were discussed and agreed on by the research working group and were audited by external experts involved in the Global Matrix 4.0 [11,12].

The grade of B for the government indicator [12] implies that the country is performing fairly well in promoting physical activity participation among Filipino youth through its policies, leadership, and resource allocation. As government was one of only two indicators wherein the country performed relatively well, it was seen as a potential actionable area to better promote physical activity. A deeper look into the policies is, therefore, necessary to better understand their breadth in terms of physical activity promotion. A previous study mainly reported on the number of policies, their descriptions and rubric scores, and the overall implications of the Report Card scores [12]. This current study aimed to do a deeper evaluation of the scope of these policies, and in the process, check for updates in policies.

## 2. Materials and Methods

This current study followed the same procedures conducted to develop the Philippine Physical Activity Report Card [11,12]. The two members originally assigned to evaluate the government indicator were joined by three other collaborators in this current study to update the evaluation of the government indicator. The five (5) investigators/authors are academics and practitioners in the fields of physical therapy and sports science. Each of them took part in searching for relevant policies, extracting information, and evaluating the policies.

To gather existing policies, the websites of the following government agencies were searched in addition to the initially searched government databases: the Department of Public Works and Highways, the Department of Transportation, the Bangsamoro Sports Commission, and the Bangsamoro Autonomous Region in Muslim Mindanao’s (BARMM) Ministry of Health and Ministry of Basic, Higher, and Technical Education. Keywords used for searching policies were “physical activity”, “physical fitness”, “health promotion”, “disease prevention”, “physical education”, “play”, “recreation”, “playground”, “public space”, “recreational space”, “sports”, and “active transport”.

Similar to previous procedures, the obtained policies were evaluated using the AHK-Wales grading system (Table 3). The authors analyzed the scope of the obtained policies and discussed the implications to policy and practice. The overall numerical score was converted to a letter grade using the Global Matrix 4.0 grading rubric (Table 2). The resulting scores from the current study were discussed and agreed on by the authors of this paper.

## 3. Results

Seven (7) were found in addition to the previously reported ten policies [12]. Table 4 shows a summary of all 17 policies, including the year they were enacted or issued, their scope or purpose(s), and sample supporting actions. Scoring based on the AHK-Wales criteria is shown in Table 3. Considering all 17 policies and their supporting actions, the updated score for the government indicator based on the AHK-Wales scoring rubric is 83%, which is equivalent to a letter grade of A− [11]. In terms of scope, these policies were found to cover promoting physical activity mainly among students, student-athletes, persons with disabilities, and the general population in school and community settings. Sports participation and active transport are the main forms of physical activity promoted by the policies. The succeeding subsections explain the results of each criterion.

### 3.1. Number and Breadth of Policies

Based on the AHK-Wales scoring rubric [13], a total of 17 policies that reference PA merits 4 out of 5 points. The breadth of these policies was scored based on the scope of policy content including the settings and population groups the policies cover, as well as the forms of physical activity promoted and the purposes of promoting physical activity.

Sports participation (47%, *n* = 8) [14,19,25,27,28,30,32,44] and active transportation (23%, *n* = 4) [16,18,39,41] are the main forms of physical activity promoted by the policies. Other policies only broadly suggest engaging in physical activity as a strategy for health and fitness [23,33,36,38,40]. In terms of purpose, most of the policies promote physical activity for sports development (47%, *n* = 8) [14,19,25,27,28,30,32,44] and health and fitness (47%, *n* = 8) [14,23,33,36,38,39,40,41]. Others are toward improving social participation and quality of life (18%, *n* = 3) [16,18,23]. Schools (53%, *n* = 9) [14,19,23,28,32,36,38,39,40] and communities (41%, *n* = 7) [16,18,23,33,38,39,41] are the main settings where physical activity is to be promoted. In terms of population groups, only five (5) policies (29%) are specific to children and adolescents [14,28,32,36,40]. All others are not specific to any age group, but these policies were still included in evaluating the government indicator, as they did not explicitly exclude children and adolescents. Seven (7) policies (41%) target developing athletes [19,25,27,28,30,32,44], two (2) (12%) are particular to persons with disabilities [18,23], and two (2) (12%) are specific to the people of BARMM [36,44].

Given the scope of the policies, which can still be improved by including more coverage of other forms of physical activity and active lifestyle (e.g., informal play and reducing sedentary behaviors), settings (e.g., homes and public spaces), and population groups (e.g., early childhood and out-of-school youth), this criterion was graded 3 out of 5 points.

### 3.2. Supporting Actions

The number of existing physical activity-promoting actions exceeded 20. Examples of these actions that arose from each policy are shown in Table 3. Supporting actions range from implementing rules and regulations [18,23,27,32], national campaigns [34,35], operation manuals and modules [37,40,46,47], guidelines [15,17,24,42,43], and sporting programs or events [20,21,22,26,45].

### 3.3. Identified Accountable Organizations

This criterion was also assigned maximum points as each policy has identified government agencies responsible for its implementation. The Philippine Sports Commission is the main responsible agency for sports-related policies [19,25,27,28,30,32], while the Department of Education and Department of Health (DOH) are the main agencies for education- [14,25,28,32,40] and health-related policies [33,38,39], respectively. Policies on active transportation and open public spaces are jointly implemented by the DOH, the Department of Transportation, the Department of Public Works and Highways, and the Department of Interior and Local Government [16,18,42]. The National Council on Disability Affairs is the main agency in charge of ensuring the implementation of policies on disability rights [18,23]. Policies on promoting an active healthy lifestyle in the BARMM are spearheaded by agencies such as the Bangsamoro Sports Commission and the Commission on Population and Development [36,44]. All policy implementations are in coordination with local government units.

### 3.4. Identified Reporting Systems

Only nine out of the seventeen policies (53%) have identified structures for reporting their implementation [19,25,27,28,38,39,40,41,44]. Actual reports of some implemented actions are available on the responsible agency’s website (e.g., PSC, *Palarong Pambansa*) or in online file storage services (e.g., HPFS [48]). However, no actual reports were found for the other policies. This criterion was therefore graded 8 out of 15 based on the rubric.

### 3.5. Identified Funding Sources

Thirteen of the seventeen policies (76%) have identified sources for funding their implementation [14,19,23,25,27,28,30,32,33,38,39,42,44]. This percentage is equal to a grade of 15 out of 20. Funding sources are from the annual budget appropriated by the national government to the responsible agencies and from the budget of local government units.

### 3.6. Identified Monitoring and Evaluation Plans

Thirteen policies (76%) have stipulated plans for monitoring and evaluating their implementation [18,19,25,27,28,30,32,33,38,39,40,42,44], which is equal to 8 out of 10 for this criterion. For most policies, the concerned agencies are responsible for monitoring and evaluating their implementation, and some are subject to Congressional oversight (e.g., the Universal Health Care Act [38] and the Philippine Sports Training Center Act [30]).

## 4. Discussion

The purpose of this study was to analyze, in depth, the scope of physical activity-related policies and update the preliminary grade of the government indicator of the 2022 Philippine Physical Activity Report Card. Seven physical activity-related policies were added to the initial ten policies. If these policies were included in the Report Card government indicator grade, the grade would have been A- instead of B. This is largely due to the additional policies expanding the scope of policy coverage and meeting most of the criteria of the AHK-Wales scoring rubric. In terms of scope, aside from physical activity for sports development, there is now greater emphasis on physical activity for health, with the inclusion of policies that mention physical activity for the prevention of non-communicable diseases, universal health care, health promotion, and physical fitness. The promotion of active transportation is also highlighted with a number of guidelines on ensuring the safe use of different forms of active transport. The inclusion of policies for BARMM and persons with disabilities is also evidence of the breadth of Philippine physical activity policies in terms of the population covered. The provisions for funding and the implementation, monitoring, and evaluation guides identified in these policies should be maximized to ensure an active healthy Filipino youth.

However, some Filipino youth, such as non-athletes and out-of-school youth, could be overlooked by these policies. In terms of scope, half of the evaluated policies are centered on participation in organized or competitive sports. While they are valuable in promoting physical activity through sports, only athletes or population groups who are sports-inclined could be benefitting from them. Policies specific to children and adolescents are also set in schools or training centers, potentially leaving behind out-of-school youth which comprises 5.5% of Filipinos aged 5 to 17 years [49]. There are also no policies for promoting physical activity among children in the early years (i.e., younger than 5 years) despite physical activity being crucial for their development [50,51]. As the rest of the policies are not specific to any population group, it is difficult to determine the extent to which Filipino youth may be benefitting from these policies. A nationally representative study that covers all youth is necessary to help recognize and act on the differential impacts of these policies on the members of Filipino youth.

Aside from meeting recommended physical activity levels, reducing sedentary behavior is also important in developing an active healthy lifestyle [1]. However, none of the evaluated policies explicitly address the sedentary behaviors of Filipinos. The country needs to expand the scope of its policies to include reducing sedentary behavior. Toward this end, efforts could incorporate existing physical activity policies and guidelines at the local and national government levels on reducing sedentary behaviors in various settings such as home, school, transportation, healthcare, and non-home-based leisure [52]. However, consistent with a whole-of-systems approach in promoting an active lifestyle [10], effective policies will require strong shifts in social norms and culture on sitting, such as how classes are held. They will also need to emphasize the broader effects of sedentary behavior not only on health, but also on productivity, learning, and community well-being [52].

The grade improvement from B to A- widened the gap between the government indicator grade and the country’s overall physical activity score for children and adolescents, which was F [12]. The overall physical activity score was based on an earlier mentioned 2018–2019 national survey [6,8] showing that the majority of Filipino adolescents are not meeting the recommended PA levels. This implies that while there are several policies advancing physical activity in the country, evidence of translation of these policies into actual engagement in physical activity is lacking. For example, despite the Accessibility Law setting standards to ensure accessibility of infrastructures to people with disabilities [18], a study showed that designs and options for public transportation vehicles in a province in the Philippines were limited, and obstructions in sidewalks, pedestrian crossings, and footbridges were common, making them unsafe for their active transport [53].

Reasons for the policy–practice gap need to be better understood to guide further action. One of the reasons could be inadequate reporting on the implementation and impact of policies and their supporting actions because data from these can be used to inform physical activity status. For example, only one data set was comprehensive enough to be of use as a basis for evaluating the overall physical activity status of Filipino youth [12]. This points to the need to look into scaling up the monitoring and reporting of the implementation of policies and supporting actions to generate data that could inform evaluations of physical activity engagement and further actions. Reports also need to be publicly available to benefit all sectors needing access to such information.

Another potential reason worth exploring is the extent to which all policies are aligned in promoting physical activity to Filipino youth. The 17 policies were found to have no clear alignment with an overarching framework to guide physical activity promotion. While the Universal Health Care Law serves as an overarching health policy, this could be deemed too broad to be effective in providing a framework specific for physical activity promotion. Additionally, the Health Promotion Framework Strategy 2030, another overarching policy, is currently limited to promoting physical activity mainly through active transportation. The country has national physical activity guidelines [54]; however, these guidelines need to be updated and aligned with current global recommendations and standards [1,10] to be able to inform national policy and action plans. These issues are pointing to the need for a comprehensive physical activity plan that could provide guidance for well-coordinated, multi-sectoral actions for better promotion of an active healthy lifestyle at the population level [4,10]. Such a plan needs to advance various forms of physical activity (e.g., active indoor or outdoor play, minimized sedentary behavior) across multiple settings, and should be inclusive of all Filipino youth (e.g., early years, children with a disability, and marginalized youth). A comprehensive physical activity plan with a multi-sectoral or whole-of-systems approach could drive better-coordinated actions among relevant sectors, which is crucial to achieving meaningful change in valuing physical activity at the societal level [1,10].

## 5. Conclusions

Several policies promote an active healthy lifestyle among Filipino youth. However, the scope of these policies suggests the need for them to be reinforced by emphasizing other forms of physical activity aside from sports and active transport, incorporating guidelines on reducing sedentary behavior, and including all members of Filipino youth. The gap between the government indicator and the overall physical activity indicator scores points to the need to better understand the reasons for the gap, including the extent of efforts to monitor and report the implementation of policies, and extent of alignment of all policies in promoting active healthy lifestyles. Effective monitoring and reporting of the implementation of policies could help in better grasping the status of physical activity and the impact of these policies on Filipino youth. A comprehensive physical activity plan could also be beneficial for better promotion of physical activity engagement. Crucial to such a plan is adopting a coordinated, multi-sectoral approach to ensure a meaningful impact at the societal level.

## Figures and Tables

**Table 1:** Global Matrix 4.0 indicators of success in promoting physical activity ^1^.

Indicators	Definition
Overall Physical Activity	Any bodily movement produced by skeletal muscles that requires energy expenditure.
Organized Sports and Physical Activity	A subset of physical activity that is structured, goal-oriented, competitive, and contest based.
Active Play	May involve symbolic activity or games with or without clearly defined rules; the activity may be unstructured/unorganized, social, or solitary, but the distinguishing features are a playful context, combined with an activity that is significantly above resting metabolic rate; tends to occur sporadically, with frequent rest periods, which makes it difficult to record.
Active Transportation	Any form of human-powered transportation—walking, cycling, using a wheelchair, in-line skating, or skateboarding.
Sedentary Behavior	Any waking behavior characterized by an energy expenditure ≤1.5 metabolic equivalents, while in a sitting, reclining, or lying posture.
Physical Fitness	Characteristics that permit a good performance of a given physical task in a specified physical, social, and psychological environment.
Family and Peers	Any member within the family who can control or influence the physical activity opportunities and participation of children and adolescents in this environment.
School	Any policies, organizational factors (e.g., infrastructure and accountability for policy implementation), or student factors (e.g., PA options based on age, gender, or ethnicity) in the school environment that can influence the physical activity opportunities and participation of children and adolescents in this environment.
Community and Environment	Any policies or organizational factors (e.g., infrastructure and accountability for policy implementation) in the municipal environment that can influence the PA opportunities and participation of children and adolescents in this environment.
Government	Any governmental body with authority to influence physical activity opportunities or participation of children and adolescents through policy, legislation, or regulation.

^1^ Adapted with permission from Aubert et al., 2022, Global Matrix 4.0 Physical Activity Report Card Grades for Children and Adolescents: Results and Analyses From 57 Countries, Journal of Physical Activity and Health Volume 19, Issue 11, Pages 700–728, https://dx.doi.org/10.1123/jpah.2022-0456. © Human Kinetics, Inc. [11].

**Table 2:** Global Matrix 4.0 grading rubric ^1^.

Grade	Interpretation
A+	94–100%
A	Succeeding with a large majority of children and adolescents (87–93%)
A−	80–86%
B+	74–79%
B	Succeeding with well over half of children and adolescents (67–73%)
B−	60–66%
C+	54–59%
C	Succeeding with about half of children and adolescents (47–53%)
C−	40–46%
D+	34–39%
D	Succeeding with less than half but some children and adolescents (27–33%)
D−	20–26%
F	Succeeding with very few children and adolescents (<20%)
INC	Incomplete due to insufficient or inadequate information to assign a grade

^1^ Adapted with permission from Aubert et al., 2022, Global Matrix 4.0 Physical Activity Report Card Grades for Children and Adolescents: Results and Analyses From 57 Countries, Journal of Physical Activity and Health Volume 19, Issue 11, Pages 700–728, https://dx.doi.org/10.1123/jpah.2022-0456. © Human Kinetics, Inc. [11].

**Table 3:** Evaluation of Philippine policies based on the AHK-Wales scoring rubric ^1^.

Criterion	Description/Scoring	Score and Justification
Number and breadth of relevant policies	Scored based on the number of policies/strategies/action plans that reference physical activity, and the breadth of the policies, i.e., the number of sectors covered in the included policies. Maximum 10 points: Number (5) + Breadth (5)	7/10 17 policies in total (4/5) Forms of physical activity are mainly sports participation and active transport; purposes are mainly for sports development and health and fitness; most policies do not specify the age group (3/5)
Identified supporting actions	Scored based on the number of policies with specific actions that promote physical activity. Each strategic document is scored a point. (Maximum of 20 points).	20/20 There are more than 20 actions supporting physical activity. (see Table 2)
Identified accountable organizations	Scored based on the proportion of policies with specific organizations identified as responsible for implementing actions. (Maximum 25 points)	25/25 Each policy has at least one specified accountable agency or organization.
Identifiable reporting structures	Scored based on the proportion of policies with explicit systems for reporting implementation of policy actions. (Maximum 15 points)	8/15 Only 9 out of the 17 policies (53%) have specified reporting structures.
Identified funding	Scored based on the proportion of policies that explicitly identified funding sources to support delivery of actions. (Maximum 20 points)	15/20 76% or 13 out of 17 policies have explicit references to sources of funding to support the implementation of the policy
Monitoring and evaluation plan	Scored based on the proportion of policies with explicit systems for monitoring and evaluation of policy progress and impact. (Maximum 10 points)	8/10 76% or 13 out of 17 policies have explicit references to plans for monitoring and evaluating the policy implementation.
Overall Score		83/100 or 83% = A−

^1^ Ward et al., The AHK-Wales Report Card 2018: Policy Measures - is it possible to ‘score’ qualitative data?, Health Promotion International, 2020, Volume 36, Issue 4, Pages 1151–1159, by permission of Oxford University Press [13].

**Table 4:** Summary of physical activity promoting policies in the Philippines ^1^.

Policy Title	Year Enacted or Issued	Scope or Purpose	Sample Supporting Actions
*Previously reported*			
The Schools Physical Education and Sports Development Act of 1969	1969	Integration of PE and sports development programs in all schools in the Philippines [14]	K to 12 Curriculum Guide: Physical Education [15]
Presidential Decree No. 1216	1977	Provision of open spaces, roads, alleys, and sidewalks to create and maintain a healthy environment in human settlements to enhance the quality of life of the residents [16]	Guidelines on the Creation and Use of Parks and Public Open Spaces [17]
Accessibility Law	1983	Setting minimum requirements and standards to make buildings, facilities, and utilities for public use accessible to persons with disabilities [18]	Accessibility Law Implementing Rules and Regulations [18]
The Philippine Sports Commission Act	1990	Creation of the Philippine Sports Commission to promote PE and encourage and sustain the development of sports in the country to foster physical fitness, self-discipline, teamwork, and excellence for the development of a healthy and alert citizenry [19]	*Batang Pinoy* (Philippine Youth Games) [20] *Laro’t Saya* sa Parke (park-based and family-oriented sports-for-all program) [21] Pilipinas Para Games [22]
Magna Carta for Disabled Persons	1991	Granting of rights and privileges to persons with disabilities by adopting policies to ensure the rehabilitation, self-development, and self-reliance of persons with disabilities, and developing their skills and potentials to enable them to compete favorably for available opportunities [23]	Implementing Rules and Regulations [23] Policy Guidelines on the Provision of Educational Programs and Services for Learners with Disabilities in the K to 12 Basic Education Program [24]
The Palarong Pambansa Act of 2013	2013	Institutionalization of the *Palarong Pambansa* (National School Games) as the premier national sporting event of the country as a venue for talent identification, selection, and recruitment of student-athletes [25]	*Palarong Pambansa* [26]
The National Athletes and Coaches Benefits and Incentives Act	2015	The promotion of excellence in sports by looking after the welfare of national athletes and coaches competing for the country and by providing benefits and incentives for national athletes and other athletes who bring honor and recognition to the country [27]	Implementing Rules and Regulations [27]
Implementing Guidelines on the Special Program in Sports	2015	Creation of a Special Program in Sports to enhance the athletic potential of talented students in different sports disciplines, and achieve school-sport balance [28]	Implementation of the Special Program in Sports in a national high school [29]
The Philippine Sports Training Center Act	2019	Establishment of the Philippine Sports Training Center to promote and develop sports in the country, and achieve excellence in international sports competitions [30]	Philippine Sports Training Center in Bataan [31]
The National Academy of Sports	2020	Establishment of the National Academy of Sports System to develop the athletic skills and talents of students in world-class sports facilities that are at par with international standards; the school system will include para-athletes [32]	Implementing Rules and Regulations [32]
*Newly obtained*			
The national policy on strengthening the prevention and control of chronic lifestyle-related non-communicable diseases	2011	Development and promotion of an integrated and comprehensive program on the prevention and control of lifestyle-related diseases in the country, wherein promoting physical activity is one of the strategies to prevent and control non-communicable diseases [33]	Pilipinas Go4Health (national campaign on healthy lifestyle against non-communicable diseases) [34] *Wais Magpapawis* (campaign on being active during the pandemic) [35]
Fatwa on the Model Family in Islam	2015	Arriving at a consensus and resolving and providing practical guidance on family-related issues, including problems related to health among youth, consistent with Shari’ah or Islamic Law to safeguard the wellbeing of individuals and society [36]	Comprehensive Gender and Health Education for Youth: Instructional Modules [37]
The Universal Health Care Act	2019	The protection and promotion of the right to health of all Filipinos, mandating agencies to address public health problems by, among others, promoting a healthy lifestyle through physical activity, proper nutrition, injury prevention, etc [38]	Health Promotion Framework Strategy [39]
The Revised Physical Fitness Test Manual	2019	Incorporating physical fitness as an essential component of the physical education and school sports program from Grades 4 through 10 [40]	Physical Fitness Test Manual [40]
Guidelines on the Proper Use and Promotion of Active Transport During and After the COVID-19 Pandemic	2020	Provision of guidance for the promotion and safe use of active transport during and after the pandemic [41]	Guidelines and Protocols for Active Transportation and Light Mobility Vehicles [42] Prescribing Guidelines on the Design of Bicycle Facilities Along National Roads [43]
The Bangsamoro Sports Commission Act of 2020	2020	Institutionalizing sports development in the BARMM by providing leadership and camaraderie among the people of Bangsamoro, formulating policies and setting the priorities and direction of all regional sports promotion activities, embarking on initiatives to develop athletic programs, and providing adequate facilities and support for Bangsamoro athletes [44]	Reviving indigenous games [45]
Health Promotion Framework Strategy 2030	2021	Provision of the framework, direction, and strategies for the planning, development, and implementation of health promotion policies, programs, plans, and activities, with physical activity as one of the priority areas for health promotion [39]	Health Promotion Playbook Modules [46] Manual of Procedures for the Operationalization of the Health Promotion Framework Strategy in Province- and City-wide Health Systems [47]

^1^ A portion of this table was adapted with permission from Cagas et al. (DOI:10.1016/j.jesf.2022.10.001) [12]. Note: BARMM—Bangsamoro Autonomous Region in Muslim Mindanao and PE—physical education.

## Data Availability

The data that support the findings of this study are available from the corresponding author, Y.Y.P., upon reasonable request.
